# Multiple Fatigue Failure Behaviors and Long-Life Prediction Approach of Carburized Cr-Ni Steel with Variable Stress Ratio

**DOI:** 10.3390/ma10091084

**Published:** 2017-09-14

**Authors:** Hailong Deng, Wei Li, Hongqiao Zhao, Tatsuo Sakai

**Affiliations:** 1 School of Mechanical Engineering, Beijing Institute of Technology, Beijing 100081, China; deng_hl@126.com (H.D.); zhaohonqiao@gmail.com (H.Z.); 2 College of Mechanical Engineering, Inner Mongulia University of Technology, Hohhot 010051, China; 3 College of Science and Engineering, Ritsumeikan University, Kusatsu 5258577, Japan; sakai@ed.ritsumei.ac.jp

**Keywords:** carburized steel, stress ratio, interior failure, initiation and growth, life prediction

## Abstract

Axial loading tests with stress ratios *R* of −1, 0 and 0.3 were performed to examine the fatigue failure behavior of a carburized Cr-Ni steel in the long-life regime from 10^4^ to 10^8^ cycles. Results show that this steel represents continuously descending *S-N* characteristics with interior inclusion-induced failure under *R* = −1, whereas it shows duplex *S-N* characteristics with surface defect-induced failure and interior inclusion-induced failure under *R* = 0 and 0.3. The increasing tension eliminates the effect of compressive residual stress and promotes crack initiation from the surface or interior defects in the carburized layer. The FGA (fine granular area) formation greatly depends on the number of loading cycles, but can be inhibited by decreasing the compressive stress. Based on the evaluation of the stress intensity factor at the crack tip, the surface and interior failures in the short life regime can be characterized by the crack growth process, while the interior failure with the FGA in the long life regime can be characterized by the crack initiation process. In view of the good agreement between predicted and experimental results, the proposed approach can be well utilized to predict fatigue lives associated with interior inclusion-FGA-fisheye induced failure, interior inclusion-fisheye induced failure, and surface defect induced failure.

## 1. Introduction

In order to satisfy the demand for mechanical parts or components with high safety and reliability in long-term service, fatigue properties of materials in the long-life regime beyond 10^7^ cycles have been drawing increasing attention because of their peculiarities [1,2]. For high strength steels [3], titanium alloys [4], cast irons [5], etc., one of the most typical failure features in the long-life regime beyond 10^7^ cycles is reflected in the change of the crack initiation mechanism from surface to interior, and this change results in the presence of “duplex or stepwise *S-N* characteristics” [3]. Although the effects of some factors such as load type [6,7], environment [8], defect size [6,9], and surface condition [10] on fatigue properties of materials in the long-life regime beyond 10^7^ cycles have been investigated, the understanding of the failure mechanisms and the relevant life prediction approaches still need to be further studied.

The conventional carburizing process is still an effective surface hardening technology for low alloy steel or low carbon steel. However, studies show that sometimes it cannot improve the fatigue strength or life of alloy steel since the probability of interior failure increases [11,12]. The interior failure of steel is often related to metallurgical defects such as inclusions, and a propagating crack shaped like a fisheye is observable [3,6,8,9,10,11,12]. In particular, a region with rough morphology can occur in the vicinity of the inclusion if the number of loading cycles is larger than about 10^6^ cycles. This special region is named as “fine granular area (FGA)” by coauthor Sakai [3]. Based on the theories of dislocation movement and irreversible slip, the FGA formation process can be divided into three stages [3,13]: (1) the formation of a fine granular layer; (2) the nucleation and coalescence of micro-debondings; (3) the completed formation of the FGA. In addition, some other theories such as “depressive decohesion of spherical carbide” [14], “hydrogen embrittlement assisted cracking” [15], “cyclic compression between crack faces” [16], “grain refinement and local stress decreasing” [17] and “nanograin refinement under negative stress effect” [18] have also been tried to elucidate the formation mechanism of FGA at a certain condition. However, the FGA formation mechanism is not yet well understood.

According to small crack theory, the √area model associated with material hardness and defect size was developed to evaluate the fatigue strength of material with interior defect-induced failure [19]. On this basis, some empirical models associated with the FGA size [20,21], fisheye size [11], equivalent crack growth rate [22], and tensile strength [23] were proposed to estimate the fatigue life or strength of a material, and to some extent they reflect the failure mechanisms of materials in the long-life regime beyond 10^7^ cycles. However, it is known that the fatigue process consists of the crack initiation process and the crack growth process. Especially in the long-life regime with relatively low stress, crack initiation should be taken into account in the total fatigue process. Unfortunately, the relevant life prediction models or methods associated with crack initiation are so far few.

In this study, the long-life fatigue properties of a carburized Cr-Ni steel with variable stress ratios were experimentally investigated under axial loading. Based on the discussion of *S-N* characteristics, failure mechanism, crack size characteristics, and stress intensity factor (SIF) at the crack tip, the long-life prediction models associated with different failure mechanisms are proposed.

## 2. Material and Experimental Method

### 2.1. Material and Specimen

**Figure 1:**
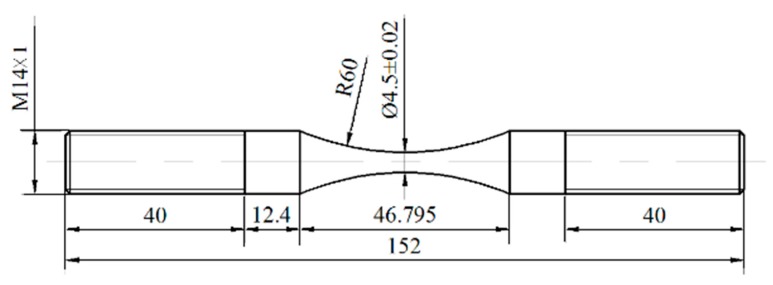
Shape and dimensions of specimen (units: mm).

The material investigated in this study was a Cr-Ni steel for the driving shaft of automotives. Its chemical composition (mass percentage) is: 0.16 C, 0.37 Si, 0.60 Mn, 0.035 S, 0.035 P, 1.65 Cr, and 3.65 Ni. First, specimens were machined into the shape of an hourglass from the annealed steel bar with a diameter of 16 mm, and then the round-notch surfaces of specimens were polished by abrasive papers with grits from p600 to p2000 in the direction parallel to the specimen axis. The final shape and dimensions of the specimen are shown in Figure 1, where the diameter of the minimum-cross-section and the radius of round-notch are 4.5 mm and 60 mm, respectively. The estimated stress concentration factor of the specimen is about 1.02 according to the stress concentration handbook [24].

### 2.2. Carburizing and Microstructure

The vacuum furnace was first filled with fresh acetylene gas by a rotary pump at a pressure of 1 Pa. Then, the specimens were put into the chamber of the vacuum furnace and the pressure of the vacuum furnace was raised to 650 Pa. Next, the furnace was heated to 880 °C and held for 0.5 h or 1 h soaking time, and then heated to the carburizing temperature of about 945 °C and held for 30 min. After the carburizing process, specimens were quenched and tempered as follows: (1) first quenching: 650 °C × 4 h + air cooling; (2) secondary quenching: 800 °C × 3 h + oil cooling; (3) tempering: 150 °C × 5 h + air cooling.

**Figure 2:**
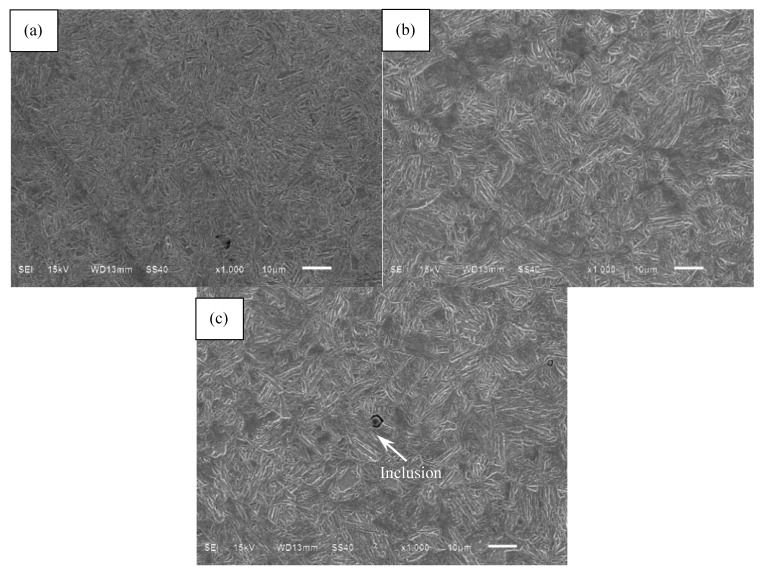
Microstructure observation of carburized Cr-Ni steel: (**a**) Microstructure in the carburized layer; (**b**) Microstructure in the core region; (**c**) Inclusion.

The observed microstructure of the heat-treated steel by using a scanning electronic microscope (SEM) is shown in Figure 2. The microstructure in the carburized layer is shown in Figure 2a, which is apparently different from that in the matrix region shown in Figure 2b. The former structure is relatively dense and consists of needle-shaped martensites and partial residual austenites with high carbon, but the latter structure is comprised of the lath martensites with low carbon. Furthermore, some non-metallic inclusions can be found in the microstructure, as shown in Figure 2c. Based on the analysis with energy dispersive X-ray spectrometer (EDS), the chemical composition of inclusion is Al_2_O_3_. In accordance with reference [25], the shear modulus and Poisson’s ratio of inclusion, *μ*_inc_ and *υ*_i_ are around 156 GPa and 0.25, respectively.

### 2.3. Mechanical Properties and Residual Stress

Based on the monotonic tension test by using MTS 809 testing system, the tensile strength, yield strength, shear modulus, and Poisson’s ratio of carburized Cr-Ni steel, *σ*_b_, *σ*_y_, *μ*_m_, and *ν*, are measured to be around 1780 MPa, 1490 MPa, 80.4 GPa, and 0.3, respectively.

**Figure 3:**
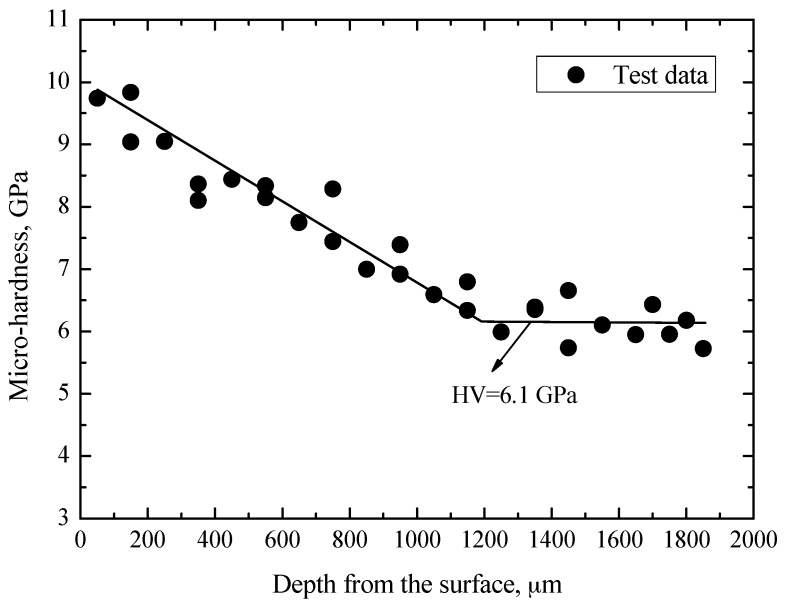
Distribution of micro-hardness.

Along the direction from the edge to the center of the cross-section of the specimen, the Vickers hardness (HV) of carburized layer and matrix region was measured by using a Nano-indenter G200 (Agilent Technologies, Santa Clara, CA, USA), and its distribution as a function of the depth from the surface is shown in Figure 3. Apparently, the values of HV first have a decreasing tendency with the increasing of the layer depth, and then reach a constant value at a depth exceeding about 1200 μm. Basically, the values of HV are about 9.9 GPa at the near surface and are about 6.1 GPa in the matrix. Thus, it can be concluded that the thickness of the carburized layer is about 1200 μm.

Based on the common sin^2^*ψ* method associated with the angle changing of incidence of Cr Kα rays, the residual stress on the round-notch surface of carburized Cr-Ni steel was measured along the axis of the specimen by a TEC 4000 X-ray diffraction system (Technology for Energy Corporation, Knoxville, TN, USA). During the test, the tube voltage was 30 kV and the tube current 6.7 mA. The maximum compressive residual stress occuring on the surface is about 268 MPa by averaging the values of four measurement points on the surface.

### 2.4. Fatigue Testing Method

By using an axial-type high frequency fatigue testing machine with about 100 Hz, fatigue tests were performed in air at room temperature, and the testing life range was about 10^4^–10^8^ cycles. The applied stress ratio *R* involves −1, 0 and 0.3. It confirms that the tested specimen has no “self-heat” phenomenon during the experiment. After the experiment, all the fracture surfaces were carefully observed by using SEM, especially the crack initiation sites and mechanisms.

## 3. Results and Discussion

### 3.1. S-N Characteristics

**Figure 4:**
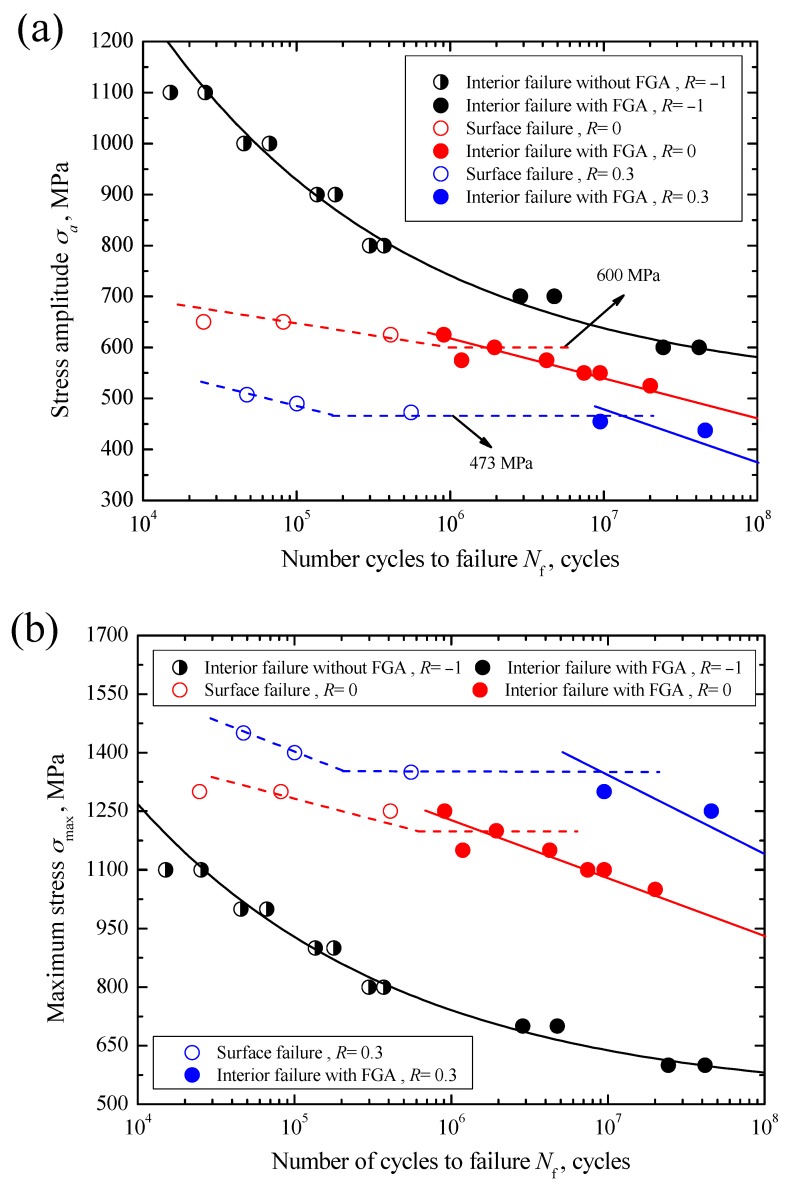
*S-N* curves of carburized Cr-Ni steel under stress ratios of −1, 0 and 0.3: (**a**) *σ*_a_ versus *N*_f_; (**b**) *σ*_max_ versus *N*_f_.

The data of applied stress amplitude *σ*_a_ versus fatigue life *N*_f_ for carburized Cr-Ni steel under axial loading with stress ratios of −1, 0 and 0.3 in the life region of 10^4^–10^8^ cycles are shown in Figure 4a. Under *R* = 0 and 0.3, by means of SEM observation of the crack initiation sites, fatigue failures of specimens can be divided into two modes: surface failure and interior failure. The surface failure mainly occurs at a relatively high stress region with a short life below 5 × 10^5^ cycles, whereas the interior failure mainly occurs at a relatively low stress region with a long life beyond about 10^6^ cycles. This result is very similar to the conventional results of some high strength steels in the long-life regime [3,6,7,9]. Approximately, duplex *S-N* characteristics are presented. Thus, herein two *S-N* curves are plotted to represent the *S-N* characteristics of carburized Cr-Ni steel associated with surface and interior failures under *R* = 0 or 0.3, indicated by dashed and solid lines in Figure 4a, respectively. Moreover, for surface failure under *R* = 0 or 0.3, a critical stress corresponding to around 10^6^ cycles can be roughly determined, as indicated by the horizontal part of the dashed line in Figure 4a, and below which surface failure cannot occur. It can be defined as the surface fatigue limit, *σ*_w-s_. Corresponding to *R* = 0 and 0.3, the values of *σ*_w-s_ are evaluated to be 600 MPa and 473 MPa, respectively. Conversely, for interior failure, the carburized Cr-Ni steel shows continuously descending *S-N* characteristics under these two stress ratios. Herein the stress amplitude at 10^8^ cycles is approximately defined as the interior fatigue limit, *σ*_w-i_, the values of *σ*_w-i_ are evaluated to be about 455 MPa under *R* = 0 and 370 MPa under *R* = 0.3 by using fitted lines, respectively.

Differing from the fatigue failures under *R* = 0 and 0.3, fatigue failure under *R* = −1 only corresponds to the interior failure whether in the short or long life region. Especially at a stress amplitude of 1100 MPa, i.e., at the maximum stress of 1100 MPa, even if the applied maximum stress nearly approaches yield strength, interior failure still occurs. In consideration of the appearance of FGA or not, the interior failure can be further subdivided into interior failure without FGA and interior failure with FGA. The former mainly occurs in the short life region of below 5 × 10^5^ cycles, whereas the latter mainly occurs in the long life region of more than 10^6^ cycles. Actually, under *R* = 0 and 0.3 the relevant interior failures only correspond to interior failure with FGA. Overall, the carburized Cr-Ni steel represents the constantly decreasing *S-N* characteristics under *R* = −1. Thus, a single *S-N* curve is plotted to represent its *S-N* characteristics under *R* = −1, as indicated by a solid black line in Figure 4a. The value of *σ*_w-i_ under *R* = −1 is evaluated to be about 580 MPa by using the fitted line. From Figure 4a, it can be seen that the applied stress amplitudes are scattered in the ranges of 600–1100 MPa under *R* = −1, 550–650 MPa under *R* = 0 and about 438–508 MPa under *R* = 0.3, respectively. Obviously, the allowable applied range of stress amplitude tends to decrease with increasing stress ratio. This is mainly attributed to the effect of the allowable applied maximum stress that should be less than the yield strength of the material. At a given stress amplitude, also it can be seen from Figure 4a that the fatigue life tends to decrease with the increasing stress ratio regardless of the failure mode.

On the other hand, the data of maximum stress *σ*_max_ versus *N*_f_ and the relevant *S-N* curves associated with the surface failure or interior failure under these three stress ratios are shown in Figure 4b. It can be seen from this figure that at a certain maximum stress level, the fatigue life tends to increase with increasing stress ratio regardless of failure mode. Also, the separation among *S-N* curves with the same failure mode is distinct. Especially, the *S-N* curve with the interior failure under *R* = 0.3 is shifted to the long-life regime beyond 10^7^ cycles. Combined with the results in Figure 4a, therefore, it can be concluded that the fatigue life or strength of material in the long-life regime is not only determined by the stress amplitude, but also determined by the maximum stress.

In addition, with increasing stress ratio from −1 to 0.3, the failure probability for interior failure decreases and that for surface failure increases. That is, the life ranges for the interior failure are about 10^4^–10^8^ cycles under *R* = −1, about 10^6^–10^8^ cycles under *R* = 0 and about 10^7^–10^8^ cycles under *R* = 0.3, respectively. The reason is that, besides the effects of the maximum stress and stress amplitude, the failure mode of carburized Cr-Ni steel under axial loading is also affected by the carburized layer with higher hardness and compressive residual stress. It is well known that the compressive residual stress can restrain crack initiation. Under the condition of tension-tension, the allowably applied maximum tensile stress tends to increase with increasing stress ratio, and it can overcome the influence of the compressive residual stress as well as promote crack initiation from the surface. However, under the condition of tension-compression, due to the smaller tensile stress and the existence of compressive stress, the crack is more easily initiated from the interior region. Also, the high hardness can restrain crack initiation from the surface under axial stress.

### 3.2. Constant Life Diagram

The Goodman equation was used to represent the effect of stress ratio *R* or mean stress *σ*_m_ on the fatigue strength of carburized Cr-Ni steel under axial loading, given by [26]

(1) $$\frac{\sigma_{a}}{\sigma_{-1}}+\frac{\sigma_{m}}{\sigma_{b}}=1$$

where *σ*_−1_ is the fatigue strength at a given fatigue life under *R* = −1. Based on the basic relation among *σ*_m_, *R* and *σ*_a_, Equation (1) can be rewritten as:

(2) $$\sigma_{a}=\sigma_{-1}\left(\frac{\sigma_{b}\left(1-R\right)}{\sigma_{b}\left(1-R\right)+\sigma_{-1}\left(1+R\right)}\right)\;\;\;\;\;-1\leq R<1$$

**Figure 5:**
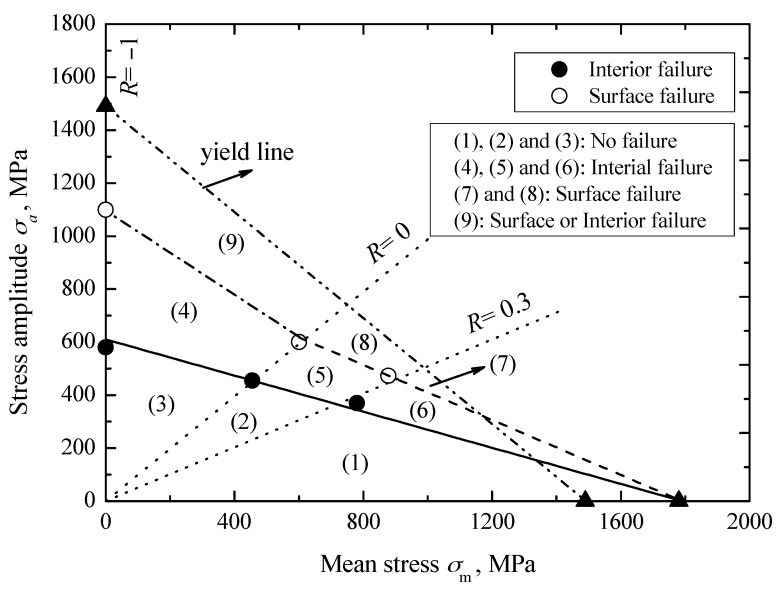
Constant life diagram of carburized Cr-Ni steel with two failure modes.

Based on Equation (2) and the evaluated values of *σ*_w-i_ under stress ratios from the *S-N* diagram in Figure 4a, the relationship between interior fatigue strength and mean stress can be established by using the least square method, as indicated by a solid line in Figure 5. This line corresponds to the constant life line with interior failure at *N* = 10^8^ cycles, and is given by:

(3) $$\sigma_{w-i}=610\left(\frac{1780\left(1-R\right)}{1780\left(1-R\right)-610\left(1+R\right)}\right)\;\;\;\;\;-1\leq R<1$$

where the values of *σ*_b_ and *σ*_−1_ at *N* = 10^8^ cycles are 1780 MPa and 610 MPa, respectively. Similarly, combined with the evaluated values of *σ*_w-s_ from the *S-N* diagram, the relationship between surface fatigue strength and mean stress is established, as indicated by a dash line in Figure 5. However since there is no surface failure data under *R* = −1, so the stress amplitude *σ*_a_ at 1100 MPa is herein assumed to be the transformation stress from surface failure to interior failure under *R* = −1, i.e., the surface failure limit at *N* = 10^6^ cycles. Thus, the relationship between surface fatigue strength and mean stress, corresponding to the stress ratio from −1 to 0, can be established, as indicated by a dash-dot line in Figure 5. The relevant constant life lines for the surface failure at *N* = 10^6^ cycles, can be given by:

(4) $$\sigma_{w-s}=\{\begin{array}{c} 930\left(\frac{1780\left(1-R\right)}{2710-850R}\right)\;\;\;\;\;\;\;\;\;\;\;\;0\leq R<1;\\ 1100\left(\frac{1320\left(1-R\right)}{2420-220R}\right)\;\;\;\;\;\;\;-1\leq R<0; \end{array}$$

Obviously, it can be seen from Figure 5 that fatigue strength has a decreasing tendency with increasing mean stress, and the decreasing rate for the surface fatigue strength is faster than that for the interior fatigue strength. Moreover, at a certain mean stress, with the decreasing of the stress ratio from 0.3 to −1, fatigue strength tends to increase regardless of failure mode.

Furthermore, the yield criterion can be expressed as [26]

(5) $$\frac{\sigma_{a}}{\sigma_{y}^{\prime }}+\frac{\sigma_{m}}{\sigma_{y}}=1$$

where $\sigma_{y}^{\prime }$ is the cyclic yield strength, but it can be replaced by *σ*_y_ as an approximation [26]. A thick dash-dot-dot line in Figure 5 indicates the relevant yield line. Therefore, combined with the established constant life lines for surface and interior failures, as well as the constant stress ratio lines for *R* = 0 and 0.3 indicated by dot lines in Figure 5, the total zone under this yield line can be divided into nine small zones. If the applied stress amplitude and mean stress fall in the zones of 1, 2, and 3, the fatigue failure and yield phenomenon do not occur within the 10^8^ cycles. If they fall in the zones of 4, 5, and 6, the interior failure is the predominant failure mode and surface failure almost does not occur. If they fall in zones 7 and 8, surface failure is predominant and interior failure almost does not occur. If they fall in zone 9, both the surface failure and the interior failure all can occur. However, it should be noted that with the decrease of tensile mean stress, the probability for interior failure tends to increase.

### 3.3. Crack Initiation Mechanism

**Figure 6:**
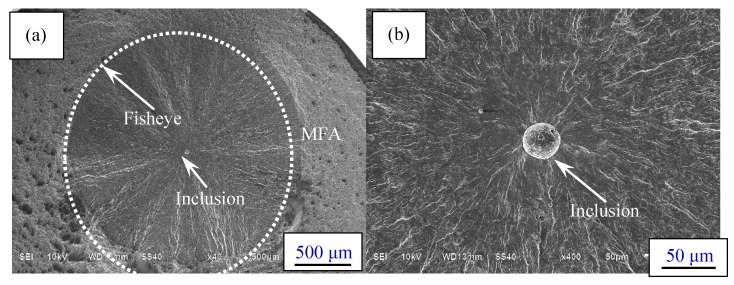

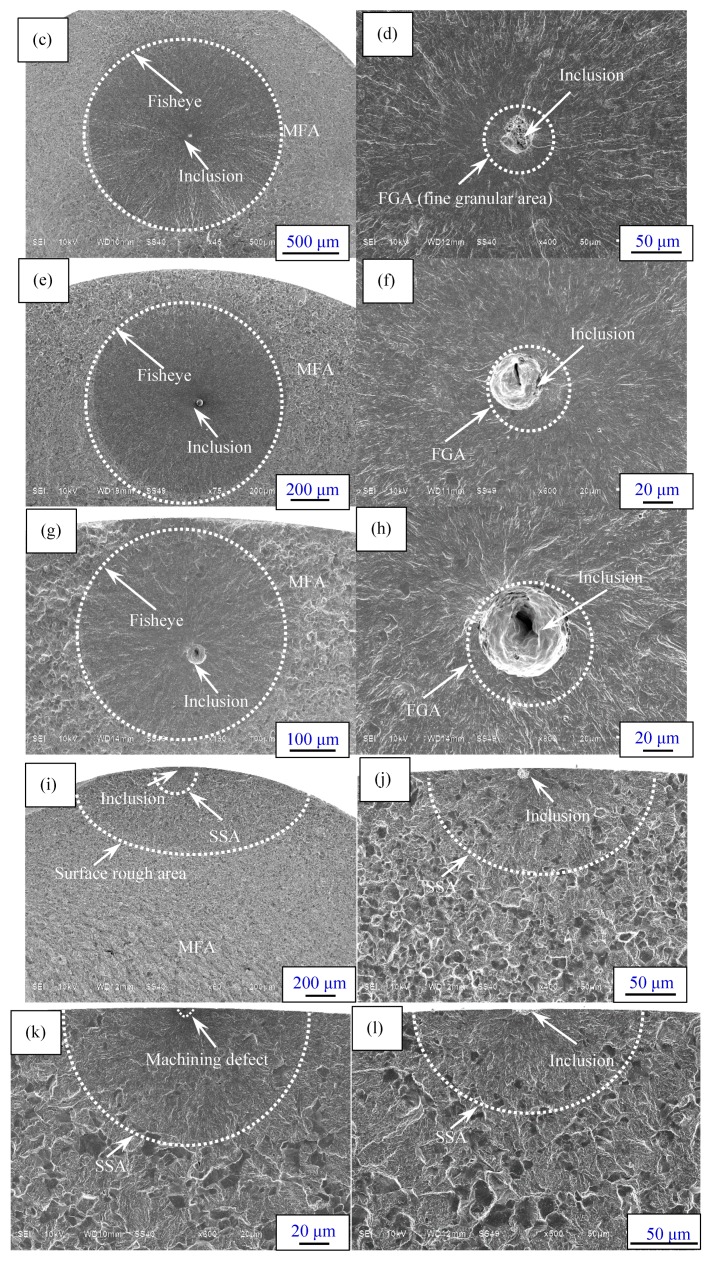
Observation of fracture surfaces: (**a**) Fisheye (*σ*_a_ = 800 MPa, *N*_f_ = 370,800 cycles, *R* = −1); (**b**) Inclusion without fine granular area (FGA) (*σ*_a_ = 575 MPa, *N*_f_ = 4,254,700 cycles, *R* = 0); (**c**) Fisheye (*σ*_a_ = 700 MPa, *N*_f_ = 4,768,700 cycles, *R* = −1); (**d**) Inclusion with FGA (*σ*_a_ = 700 MPa, *N*_f_ = 4,768,700 cycles, *R* = −1); (**e**) Fisheye (*σ*_a_ = 575 MPa, *N*_f_ = 4,254,700 cycles, *R* = 0); (**f**) Inclusion with FGA (*σ*_a_ = 575 MPa, *N*_f_ = 4,254,700 cycles, *R* = 0); (**g**) Fisheye (*σ*_a_ = 455 MPa, *N*_f_ = 9,494,900 cycles, *R* = 0.3); (**h**) Inclusion with FGA (*σ*_a_ = 455 MPa, *N*_f_ = 9,494,900 cycles, *R* = 0.3); (**i**) Surface failure (*σ*_a_ = 650 MPa, *N*_f_ = 82,100 cycles, *R* = 0); (**j**) Inclusion (*σ*_a_ = 650 MPa, *N*_f_ = 82,100 cycles, *R* = 0); (**k**) Surface machining defect (*σ*_a_ = 600 MPa, *N*_f_ = 1,945,900 cycles, *R* = 0); (**l**) Inclusion (*σ*_a_ = 490 MPa, *N*_f_ = 100,200 cycles, *R* = 0.3).

As mentioned above, fatigue failures of specimens consist of surface failure, interior failure without FGA and interior failure with FGA. For interior failure, fatigue cracks all originate from interior non-metallic inclusions. The deformation inconsistency between the inclusion and the ambient matrix contributes to the crack initiation. Figure 6a–h present the typical fracture surfaces with interior failure under different stress ratios. The fisheyes can be observed on the fracture surfaces, and the inclusions are nearly located at the center of the fisheyes, as shown in Figure 6a,c,e,g.

Because of the inhibiting effect of the carburized layer, the inclusions which cause crack initiation are mainly confined to the core matrix region under *R* = −1. In other words, even if there is a larger inclusion in the carburizing layer, it will not induce fatigue failure. However, with the increasing stress ratio from −1 to 0.3, the occurrence probability of interior inclusion-induced crack initiation in the carburizing layer tends to increase. This is attributed to the increasing tensile stress under higher stress ratio, which will overcome the effect of the carburized layer with compressive residual stress. Moreover, it can be found that the sizes of fisheyes within the carburized layer are smaller than those in the core region due to the relatively slow crack growth rate in the carburized layer. Detailed analysis about crack sizes will be given in the following section.

FGA cannot be observed around the inclusion in the short life region of below 5 × 10^5^ cycles, as shown in Figure 5b. Otherwise, in the long life region of about more than 10^6^ cycles, it can be found under each stress ratio, as shown in Figure 6d,f,h. It is confirmed that the formation of FGA is largely related to the number of loading cycles. Maybe, at least fatigue life with about 10^6^ cycles is a necessary condition for FGA formation.

Furthermore, from Figure 6d,f,h, it can be seen that with increasing stress ratio, the FGA morphology becomes vague and the FGA size approximately tends to decrease. Especially under *R* = 0.3, the FGA is hard to distinguish. This reveals that increasing stress ratio, i.e., decreasing of compressive stress and stress amplitude, may restrain FGA formation. In general, the fracture surface without FGA can be divided into three areas: (i) inclusion; (ii) fisheye; (iii) momentary fracture area (MFA), as shown in Figure 6a. The fracture surface with FGA can be divided into four areas: (i) inclusion; (ii) FGA; (iii) fisheye, and (iv) MFA, as shown in Figure 6c or e.

For the surface induced failure, the typical fracture surfaces under different stress ratios are shown in Figure 6i–l. Fatigue cracks mainly originate from surface inclusions shown in Figure 6j,l or surface machining defects shown in Figure 6k. Herein, the surface inclusion and machining defect are all defined as surface defect (SD). Therefore, the entire fracture surface with surface failure can be roughly divided into four areas: (i) SD; (ii) surface smooth area (SSA); (iii) surface rough area; (iv) MFA, as shown in Figure 6i.

### 3.4. Discussion Based on Fractography

**Figure 7:**
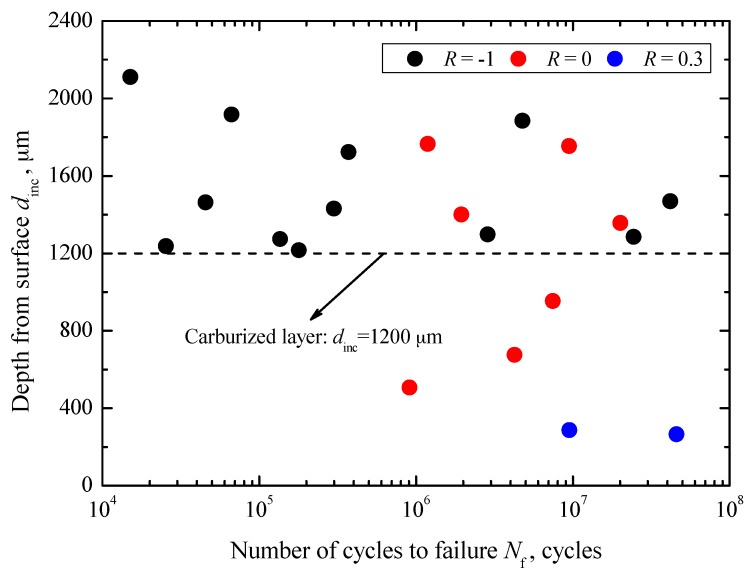
Relationship between *d*_inc_ and *N*_f_.

Based on fractography, several parameters were defined to discuss the crack geometrical characteristics. For interior failure, the parameter *d*_inc_ was defined to indicate the depth of interior inclusion from the center to the nearest edge of the fracture surface. Parameters *r*_inc_, *r*_FGA_, and *r*_fisheye_ were defined to denote the radius of inclusion, FGA, and fisheye, respectively. For surface failure, the shapes of SD and SSA were also approximately considered to be circular. Thus, *r*_SD_ and *r*_SSA_ were defined to denote the radius of SD and SSA, respectively. The relationships between *d*_inc_ and *N*_f_ under stress ratios of −1, 0 and 0.3 are shown in Figure 7.

**Figure 8:**
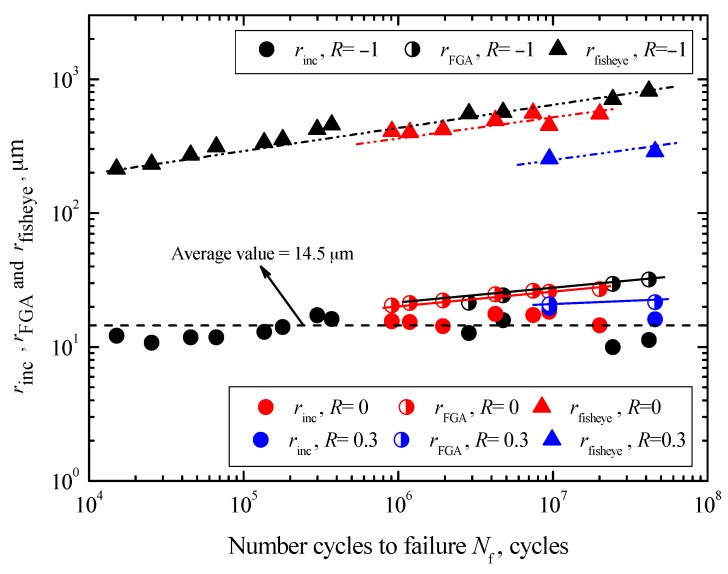
Relationships between *r*_inc_, *r*_FGA_, and *r*_fisheye_, and *N*_f_.

In general, the values of *d*_inc_ under these three stress ratios are independent of fatigue life. The values of *d*_inc_ under *R* = −1 are scattered in the range of 1216–2110 μm, which are obviously larger than the depth of the carburized layer. Under *R* = 0, the values of *d*_inc_ are scattered in the range of 506–1765 μm, some of which are less than the depth of carburized layer. Under *R* = 0.3, the values of *d*_inc_ are only scattered in the range of 265–285 μm, all less than the depth of carburized layer. Even only two data points under the stress ratio of 0.3 are obtained, but it can be found that the failure probability relating to the inclusions contained in the carburized layer is larger at a higher stress ratio. This is consistent with the results of fracture surface observation. The reason is that at positive stress ratio, only tension is applied and it will easily overcome the compressive residual stress in the carburized layer and promote crack initiation from the carburized layer. In other words, under higher stress ratio, the effect of the damage zone becomes larger. The relationships between *r*_inc_, *r*_FGA_ and *r*_fisheye_, and *N*_f_ under stress ratios of −1, 0 and 0.3 are shown in Figure 8.

First, the values of *r*_inc_ are scattered over the range between 9.9 μm and 19.6 μm, regardless of both fatigue life and stress ratio. The average value is about 14.5 μm, as denoted by a dashed black line in Figure 8. Conversely, this means that the sizes of inclusion mainly rely on the melting technique of steel. Secondly, under each stress ratio, the values of *r*_FGA_ all tend to increase with increasing *N*_f_, denoted by solid lines with different colors in Figure 8. Corresponding to a certain fatigue life, the values of *r*_FGA_ under *R* = −1 are slightly larger than those under *R* = 0, but this phenomenon is not so distinct in the log-log coordinate. However, obviously the values of *r*_FGA_ are the smallest under *R* = 0.3. Therefore, it can be concluded that the sizes of FGA tend to decrease with increasing stress ratio at a certain fatigue life. This confirms that at a given fatigue life, the higher stress ratio associated with larger *σ*_max_ and lower *σ*_a_ will promote crack growth and restrain FGA formation at the crack initiation stage. Furthermore, by defining *ρ*_FGA_ as the ratio of *r*_FGA_ to *r*_inc_, the numerical relationships between *ρ*_FGA_ and *σ*_a_ under different stress ratios can be described as

(6) $$\rho_{\mathrm{FGA}}=\frac{r_{\mathrm{FGA}}}{r_{\mathrm{inc}}}=f_{1}\left(R\right)\sigma_{a}^{f_{2}\left(R\right)}$$

and

(7) $$f_{1}\left(R\right)=18,614.93+3.03\times 10^{16}R+3.03\times 10^{16}R^{2}$$

(8) $$f_{2}\left(R\right)=-1.49-11.12R-13.47R^{2}$$

where *f*_1_(*R*) and *f*_2_(*R*) are the functions of *R*. Based on Equation (6), the FGA size at a given stress state can be estimated if the inclusion size is known. The sizes of the fisheye also tend to increase with increasing *N*_f_ at each stress ratio, and the variation trends are indicated by doubly chained lines with different colors. The values of *r*_fisheye_ under *R* = 0 are lower than those under *R* = −1, while those under *R* = 0.3 are lowest at a certain fatigue life. Just like FGA, the sizes of fisheye tend to decrease with increasing stress ratio at a certain fatigue life.

**Figure 9:**
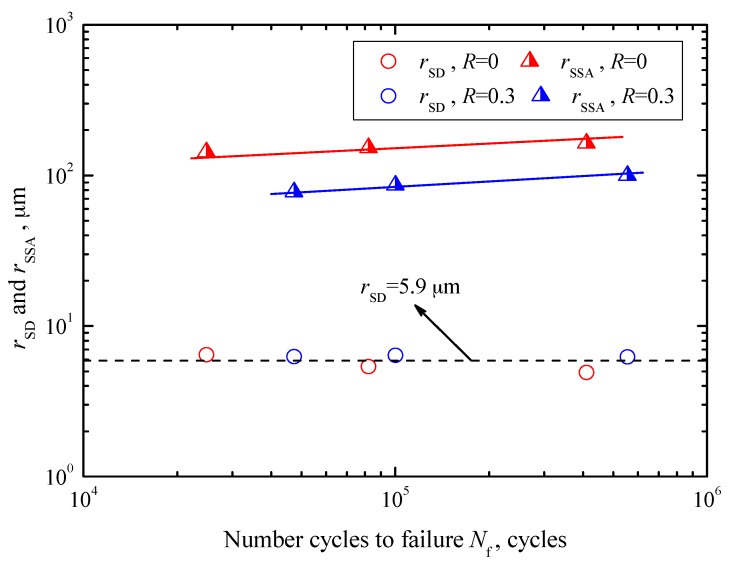
Relationships between *r*_SD_ and *r*_SSA_, and *N*_f_.

On the other hand, the relationships between *r*_SD_ and *r*_SSA_, and *N*_f_ under these three stress ratios are shown in Figure 9. The values of *r*_SD_ are scattered throughout 4.9–6.5 μm with an average value of 5.9 μm, independent of fatigue life and stress ratio, as denoted by a dashed line in Figure 9. Apparently, surface defects are mainly dependent on the machining process. Moreover, the values of *r*_SSA_ tend to increase with increasing fatigue life at each stress ratio, indicated by solid lines with different colors in Figure 9. The values of *r*_SSA_ under *R* = 0 are scattered in the range of 143.3–163.4 μm, while those under *R* = 0.3 are scatted in the range of 77.7–99.9 μm. By contrast, the sizes of SSA under *R* = 0 are obviously larger at a certain fatigue life.

### 3.5. Evaluation of Stress Intensity Factor

For interior failure, the inclusion, FGA, and fisheye all are approximately equivalent to interior circular cracks [27]. Thus, their stress intensity factor (SIF) ranges, Δ*K*_inc_, Δ*K*_FGA,_ and Δ*K*_fisheye_, are determined by [28]:

(9) $$\Delta K_{\mathrm{inc},\;\mathrm{or}\;\mathrm{FGA}\;\mathrm{and}\;\mathrm{or}\;\mathrm{fisheye}}=\frac{2}{\pi }\Delta \sigma \sqrt{\pi r_{\mathrm{inc},\;\mathrm{or}\;\mathrm{FGA}\;\mathrm{and}\;\mathrm{or}\;\mathrm{fisheye}}}$$

where ∆*σ* is the stress range. Similarly, for surface failure, the SIF ranges for the SD and SSA, Δ*K*_SD_ and Δ*K*_SSA_, are given by [28]:

(10) $$\Delta K_{\mathrm{SD}\;\mathrm{and}\;\mathrm{or}\;\mathrm{SSA}}=\frac{1.12\Delta \sigma \sqrt{\pi r_{\mathrm{SD}\;\mathrm{or}\;\mathrm{SSA}}}}{E\left(k\right)}≅\Delta \sigma \sqrt{\pi r_{\mathrm{SD}\;\mathrm{or}\;\mathrm{SSA}}}$$

where parameter *E*(*k*) is between 1 and *π*/2.

**Figure 10:**
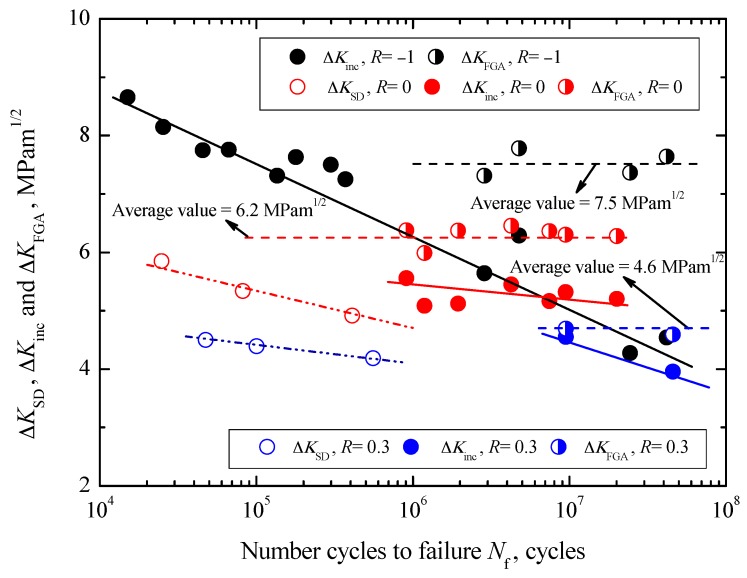
Relationships between Δ*K*_SD_, Δ*K*_inc_ and Δ*K*_FGA_, and *N*_f_.

The relationships between Δ*K*_inc_ and Δ*K*_FGA_, and *N*_f_ under three stress ratios are shown in Figure 10. First, the values of Δ*K*_inc_ are in the range of 4.3–8.6 MPa∙m^1/2^ under *R* = −1, 5.1–5.6 MPa∙m^1/2^ under *R* = 0 and 3.9–4.5 MPa∙m^1/2^ under *R* = 0.3, respectively. They all tend to decrease with increasing *N*_f_, i.e., decreasing of ∆*σ*, under each stress ratio, denoted by some solid lines in Figure 10. Moreover, at a given fatigue life the values of Δ*K*_inc_ tend to decrease with increasing stress ratio. Secondly, the values of Δ*K*_FGA_ are in the ranges of 7.3–7.8 MPa∙m^1/2^ under *R* = −1, 5.9–6.5 MPa∙m^1/2^ under *R* = 0 and 4.5–4.7 MPa∙m^1/2^ under *R* = 0.3, respectively. Approximately, they keep constant under each stress ratio, and the mean values are 7.5 MPa∙m^1/2^ under *R* = −1, 6.2 MPa∙m^1/2^ under *R* = 0 and 4.6 MPa∙m^1/2^ under *R* = 0.3, respectively. Compared with the change tendency of Δ*K*_inc_, the values of Δ*K*_FGA_ are regardless of fatigue life, but also tend to decrease with increasing stress ratio, denoted by dashed lines in Figure 10. Under *R* = −1, the values of Δ*K*_FGA_ are larger than the values of Δ*K*_inc_ at a certain fatigue life when the FGA can be observed in the vicinity of inclusion. However, the values of Δ*K*_inc_ corresponding to the nonexistence of FGA are approximately not less than the mean value of Δ*K*_FGA_ at about 7.5 MPa∙m^1/2^. Therefore, it can be assumed that the upper limit value of Δ*K*_inc_ corresponding to the formation FGA is about 7.5 MPa∙m^1/2^. In other words, FGA will not be formed if Δ*K*_inc_ is larger than 7.5 MPa∙m^1/2^. Similarly, the upper limit values of Δ*K*_inc_ corresponding to the formation FGA are evaluated to be about 6.2 MPa∙m^1/2^ under *R* = 0 and 4.6 MPa∙m^1/2^ under *R* = 0.3, respectively.

Compared with the other experimental results [3,7], the evaluated values of Δ*K*_FGA_ under *R* = −1 in this study are larger. The reason is that in these studies, the stress amplitude *σ*_a_ is used for the calculation of Δ*K* rather than the stress range. Based on the FGA formation mechanism and fracture surface morphology, Δ*K*_FGA_ at different stress ratios can be defined as the threshold values controlling stable growth of interior macroscopic crack at different stress ratios, and the fatigue behavior within the FGA is mainly governed by crack initiation.

**Figure 11:**
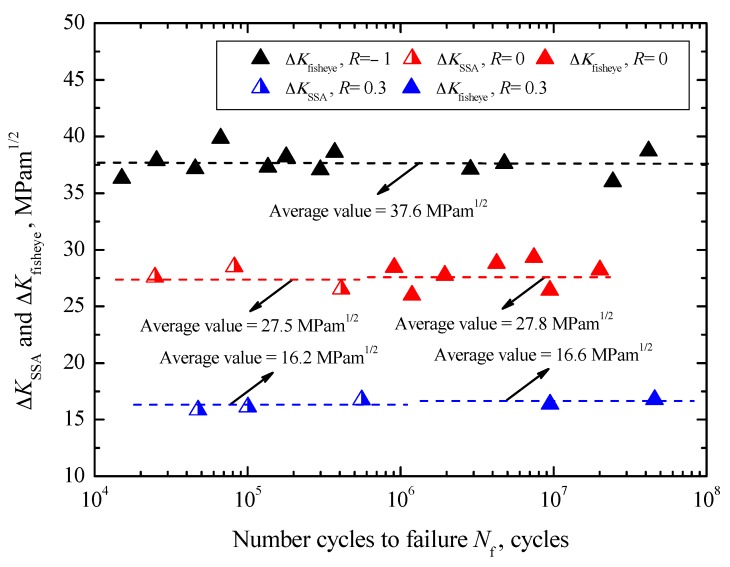
Relationships between Δ*K*_SSA_ and Δ*K*_fisheye_, and *N*_f_.

The values of Δ*K*_fisheye_ are in the ranges of 35.9–39.8 MPa∙m^1/2^ under *R* = −1, 25.9–29.3 MPa∙m^1/2^ under *R* = 0, and 16.3–16.7 MPa∙m^1/2^ under *R* = 0.3, respectively. Approximately, they also keep constant under each stress ratio, and the corresponding mean values are 37.6 MPa∙m^1/2^ under *R* = −1, 27.8 MPa∙m^1/2^ under *R* = 0 and 16.6 MPa∙m^1/2^ under *R* = 0.3, respectively. Just like Δ*K*_FGA_, the values of Δ*K*_fisheye_ are also regardless of fatigue life and tend to decrease with increasing stress ratio, denoted by dashed lines in Figure 11. Previous studies [3,7,9] had confirmed that the formation of fisheye means the beginning of the interior crack growth in an unstable manner, and ∆*K*_fisheye_ can be regarded as the threshold value referring to the condition in which a crack extends in an unstable manner without an increase in load.

On the other hand, the relationships between Δ*K*_SD_ and *N*_f_ under two stress ratios are also shown in Figure 10. The values of Δ*K*_SD_ are in the ranges of 4.9–5.8 MPa∙m^1/2^ under *R* = 0 and 4.2–4.5 MPa∙m^1/2^ under *R* = 0.3, respectively. By contrast, it can be found that under the same stress ratio, the values of Δ*K*_SD_ are slightly higher than those of Δ*K*_inc_, and the partial values are similar to the values of Δ*K*_FGA_. Moreover, just like Δ*K*_inc_, they tend to decrease with increasing *N*_f_ under each stress ratio, and to decrease with increasing stress ratio. From the viewpoint of crack growth, approximately the minimum value of Δ*K*_SD_ can be defined as the threshold value controlling surface crack growth under a given ratio, about 4.9 MPa∙m^1/2^ under *R* = 0 and 4.2 MPa∙m^1/2^ under *R* = 0.3. Correspondingly, that is the reason why the *S-N* curves of carburized Cr-Ni steel have the traditional asymptote shape under stress ratios of 0 and 0.3.

The relationship between Δ*K*_SSA_ and *N*_f_ is also shown in Figure 11. The values of Δ*K*_SSA_ are in the range of 26.5–28.5 MPa∙m^1/2^ with an average value of 27.5 MPa∙m^1/2^ under *R* = 0, while those are in the range of 15.8–16.7 MPa∙m^1/2^ with an average value of 16.2 MPa∙m^1/2^ under *R* = 0.3, denoted by dashed lines in Figure 11. Apparently, the values of Δ*K*_SSA_ tend to decrease with increasing stress ratio, regardless of fatigue life. Moreover, it should be noted that the values of Δ*K*_SSA_ are very similar to the values of Δ*K*_fisheye_ at each stress ratio. Therefore, herein Δ*K*_SSA_ can be approximately regarded as the threshold value controlling the unstable growth of surface crack.

Therefore, it can be concluded that fatigue processes for the interior failure without FGA and surface failure in a short life regime less than 5 × 10^5^ cycles are mainly dominated by crack growth from inclusion to fisheye or from SD to SSA. Conversely, the fatigue process for interior failure with FGA in the long life regime larger than 10^6^ cycles is largely governed by crack initiation within FGA.

### 3.6. Fatigue Life Modeling

#### 3.6.1. Modeling for Interior Failure with FGA

Since the inclusion and the interface are strong enough not to break at low stress in the long life regime, the plastic flow accumulates in the matrix after a time of cyclic loading. The motion of dislocation in the matrix will be blocked by inclusion, an elliptic slip band zone around the inclusion will be formed. The accumulation of dislocations impinging on the inclusion will eventually result in the inclusion debonding or cracking. The number of loading cycles for crack initiation can be obtained by equating the stored energy in the dipoles to the specific fracture energy per unit area. Based on the assumption that all the dislocation dipoles in the slip band zone contribute to the crack initiation, the crack initiation life, *N*_i_, for the interior inclusion-induced cracking can be expressed as [29]

(11) $$(\Delta \tau -2k)N_{i}^{1/2}={\left[\frac{\mu_{m}\left(\mu_{m}+\mu_{\mathrm{inc}}\right)}{\mu_{\mathrm{inc}}}\right]}^{1/2}\left(\frac{h}{h+l}\right){\left(\frac{4W_{s}}{r_{\mathrm{inc}}}\right)}^{1/2}$$

where *μ*_m_ is the shear modulus of matrix, *μ*_inc_ is the shear modulus of inclusion, *h* and *l* are semi-minor and semi-major of the slip band zone respectively, *W*_s_ is the specific fracture energy for a unit area along the slip band, Δ*τ* is the local shear stress range, and *k* is the friction stress of dislocation. However, recent researches show that not all dislocation dipoles contribute to the formation of crack, at least not immediately at crack initiation, and the crack length or size at crack initiation is related to the number of dislocations that contribute to crack formation [30]. Thus, based on the relation between the crack size at crack initiation, the number of dislocations contribute to crack formation and fracture energy, and the value of *W*_s_ can be revised as [31]

(12) $$W_{s}=\frac{\mu_{m}c}{0.005}{\left(\frac{h}{2l}\right)}^{2}$$

where *c* is defined as the crack size at crack initiation, and 0.005 is a universal value [30]. Substituting Equation (12) into Equation (11), we have

(13) $$(\Delta \tau -2k)N_{i}^{1/2}={\left[\frac{\left(\mu_{m}+\mu_{\mathrm{inc}}\right)}{0.005\mu_{\mathrm{inc}}}\right]}^{1/2}\frac{\mu_{m}h^{2}}{l\left(h+l\right)}{\left(\frac{c}{r_{\mathrm{inc}}}\right)}^{1/2}$$

By using the Taylor factor *M*, the shear stress can be converted to normal stress. In other words, the local shear stress range corresponds to normal stress (i.e., fatigue strength), the friction stress of dislocation corresponds to the limit of normal stress (i.e., the fatigue limit). By using the Taylor factor, they can be converted each other. Moreover, for better agreement with the experimental data, the exponent of *N*_i_ is generalized to a variable *α* (0 < *α* < 1) [31]. Equation (13) for *R* = −1 can be given by:

(14) $$(\sigma_{-1}-\sigma_{w\left(R=-1\right)})N_{i}^{\alpha }={\left[\frac{\left(\mu_{m}+\mu_{\mathrm{inc}}\right)}{0.005\mu_{\mathrm{inc}}}\right]}^{1/2}\frac{M\mu_{m}h^{2}}{2l\left(h+l\right)}{\left(\frac{c}{r_{\mathrm{inc}}}\right)}^{1/2}$$

where *σ*_w_ (*R* = −1) corresponds to *σ*_w-i_ for *R* = −1. If the slip band zone is assumed to expand to the whole FGA, Equation (14) is rewritten as

(15) $$(\sigma_{-1}-\sigma_{w\left(R=-1\right)})N_{i}^{\alpha }={\left[\frac{\left(\mu_{m}+\mu_{\mathrm{inc}}\right)}{0.005\mu_{\mathrm{inc}}}\right]}^{1/2}\frac{M\mu_{m}h_{\mathrm{FGA}}^{2}}{2l_{\mathrm{FGA}}\left(h_{\mathrm{FGA}}+l_{\mathrm{FGA}}\right)}{\left(\frac{c}{r_{\mathrm{inc}}}\right)}^{1/2}$$

where *h*_FGA_ and *l*_FGA_ are defined as the semi-minor and semi-major of the FGA, respectively. Since the shape of the FGA is considered to be circular, so *l*_FGA_ and *h*_FGA_ are equal in size. Thus, Equation (15) is simplified as

(16) $$(\sigma_{-1}-\sigma_{w\left(R=-1\right)})N_{i}^{\alpha }={\left[\frac{\left(\mu_{m}+\mu_{\mathrm{inc}}\right)}{0.005\mu_{\mathrm{inc}}}\right]}^{1/2}\frac{M\mu_{m}}{4}{\left(\frac{c}{r_{\mathrm{inc}}}\right)}^{1/2}$$

And the crack initiation life corresponding to different *c*-values under *R* = −1 is given by:

(17) $$N_{i}={\left[\frac{\left(\mu_{m}+\mu_{\mathrm{inc}}\right)}{0.005\mu_{\mathrm{inc}}}\right]}^{1/2\alpha }{\left(\frac{M\mu_{m}}{4(\sigma_{-1}-\sigma_{w\left(R=-1\right)})}\right)}^{1/\alpha }{\left(\frac{c}{r_{\mathrm{inc}}}\right)}^{1/2\alpha }$$

According to the test data and the sizes of inclusion and FGA, the value of *α* can be determined by using the least square method, and the value of *N*_i_ can be obtained. Next, considering the effect of mean stress or stress ratio on crack initiation life, and combining with Equation (2), the corresponding crack initiation life can be given by:

(18) $$N_{i}={\left[\frac{\left(\mu_{m}+\mu_{\mathrm{inc}}\right)}{0.005\mu_{\mathrm{inc}}}\right]}^{1/2\alpha }{\left(\frac{M\mu_{m}}{4(\eta \sigma_{a}-\sigma_{w\left(R=-1\right)})}\right)}^{1/\alpha }{\left(\frac{c}{r_{\mathrm{inc}}}\right)}^{1/2\alpha }$$

and

(19) $$\eta =\frac{\sigma_{b}\left(1-R\right)+\sigma_{-1}\left(1+R\right)}{\sigma_{b}\left(1-R\right)}$$

In addition, the FGA size is unknown before the experiment. However, based on the relationship between the inclusion size and the FGA size, described in Equation (6), the FGA size can be evaluated. Thus, the crack initiation life model corresponding to the inclusion-FGA induced failure in the long-life regime under different stress ratios can be given by:

(20) $$N_{i}={\left[\frac{\left(\mu_{m}+\mu_{\mathrm{inc}}\right)}{0.005\mu_{\mathrm{inc}}}\right]}^{1/2\alpha }{\left(\frac{M\mu_{m}}{4(\eta \sigma_{a}-\sigma_{w})}\right)}^{1/\alpha }{\left(\rho_{\mathrm{FGA}}-1\right)}^{1/2\alpha }$$

#### 3.6.2. Modeling for Interior Failure without FGA

As mentioned above, the interior inclusion-induced failure without FGA in the short life regime less than 5 × 10^5^ cycles mainly corresponds to the crack growth stage. That is, the interior crack growth life, *N*_g-int_, can be approximately considered as the total fatigue life. According to the Paris law, first, the relevant crack growth rate, (*da/dN*)_int_, can be given by

(21) $${\left(da/dN\right)}_{\mathrm{int}}=C_{\mathrm{int}}{\left(\Delta K\right)}^{m_{\mathrm{int}}}$$

where *C*_int_ and *m*_int_ are material constants, respectively. Integrating Equation (21) from the inclusion size to the fisheye size at the final fracture and combining Equation (9), then, we have

(22) $${\left(\frac{2}{\pi }\Delta \sigma \sqrt{\pi r_{\mathrm{inc}}}\right)}^{m_{\mathrm{int}}}\left(\frac{N_{g-\mathrm{int}}}{r_{\mathrm{inc}}}\right)=\frac{2}{C_{\mathrm{int}}\left(m_{\mathrm{int}}-2\right)}\left[1-{\left(\frac{r_{\mathrm{inc}}}{r_{\mathrm{fisheye}}}\right)}^{\frac{m_{\mathrm{int}}}{2}-1}\right]$$

Due to the value of *r*_inc_ being less than that of *r*_fisheye_, so Equation (22) can be reduced to

(23) $${\left(\frac{2}{\pi }\Delta \sigma \sqrt{\pi r_{\mathrm{inc}}}\right)}^{m_{\mathrm{int}}}\left(\frac{N_{g-\mathrm{int}}}{r_{\mathrm{inc}}}\right)=\frac{2}{C_{\mathrm{int}}\left(m_{\mathrm{int}}-2\right)}$$

Under *R* = −1, the value of *N*_g-int_ can be expressed as

(24) $$N_{g-\mathrm{int}}=\frac{2r_{\mathrm{inc}}}{C_{\mathrm{int}}\left(m_{\mathrm{int}}-2\right)}{\left(\frac{4}{\pi }\sigma_{-1}\sqrt{\pi r_{\mathrm{inc}}}\right)}^{-m_{\mathrm{int}}}$$

Combined with Equation (2), finally, the crack growth life model corresponding to the inclusion-fisheye induced failure under different stress ratios can be established as

(25) $$N_{g-\mathrm{int}}=\frac{2r_{\mathrm{inc}}}{C_{\mathrm{int}}\left(m_{\mathrm{int}}-2\right)}{\left(\frac{4}{\pi }\eta \sigma_{a}\sqrt{\pi r_{\mathrm{inc}}}\right)}^{-m_{\mathrm{int}}}$$

#### 3.6.3. Modeling for Surface Failure

In the same way, the surface defect-induced failure in the short life regime less than 5 × 10^5^ cycles also can be characterized from the viewpoint of crack growth. According to the Paris law, and integrating from the SD size to the SSA size and combining Equation (2), the surface crack growth life, *N*_g-sur_, corresponding to the surface SD–SSA induced failure in the relatively short regime under different stress ratios can be expressed as

(26) $$N_{g-\mathrm{sur}}=\frac{2r_{\mathrm{SD}}}{C_{\mathrm{sur}}\left(m_{\mathrm{sur}}-2\right)}{\left(\frac{4}{\pi }\eta \sigma_{a}\sqrt{\pi r_{\mathrm{SD}}}\right)}^{-m_{\mathrm{sur}}}$$

where *C*_sur_ and *m*_sur_ are material constants, respectively. Herein, the values of *C*_sur_ and *m*_sur_ are obtained by using the test data for *R* = 0 due to the data deficiency for *R* = −1.

### 3.7. Fatigue Life Prediction

**Figure 12:**
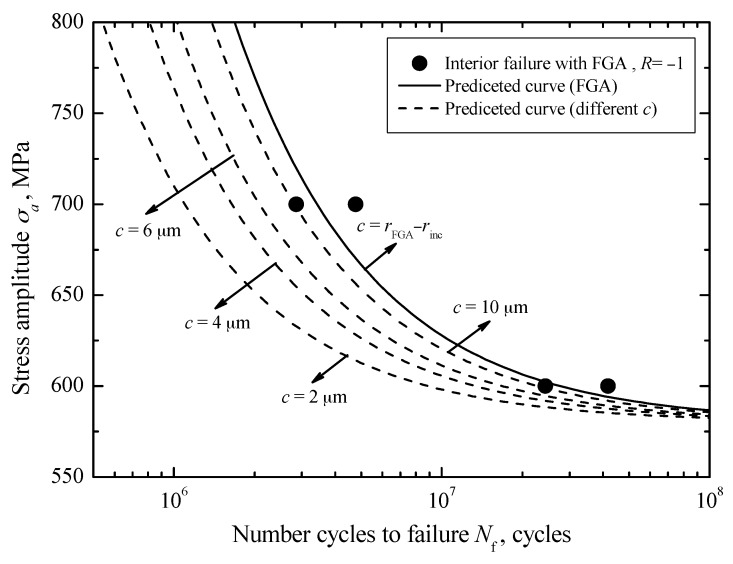
Predicted crack initiation lives at different *c*-values under *R* =−1.

**Figure 13:**
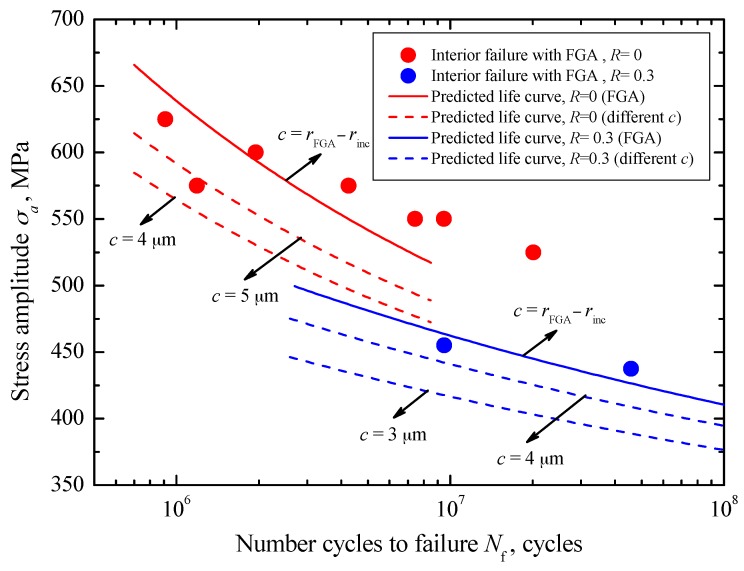
Predicted crack initiation lives at different *c*-values under *R* = 0 and 0.3.

For interior failure with FGA, by using Equation (17), the crack initiation life curves corresponding to different *c*-values under *R* = −1 can be established, as denoted by some dashed lines in Figure 12. Apparently, it can be seen that the crack initiation life has an increasing tendency with increasing crack size at a certain stress. Moreover, for a given crack size, the required number of cycles at the lower stress level is larger than that at the higher stress level, and almost approaches the total fatigue life. It should be noted that the established crack initiation life curve corresponding to the FGA size, indicated by a solid line in Figure 12, is consistent with the experimental data. This further reveals that the long life is mainly consumed in the crack initiation stage inside the FGA. Based on the results under *R* = −1 and Equation (20), the predicted crack initiation life curves corresponding to FGA sizes under *R* = 0 and *R* = 0.3 can be plotted by two solid lines as in Figure 13, respectively. Basically, the predicted curves well reflect the change trend of test data under these two stress ratios. Moreover, under each stress ratio, some crack initiation life curves corresponding to the small crack sizes less than the FGA size are plotted by the dashed lines in Figure 13. Similar results can be seen in Figure 12.

**Figure 14:**
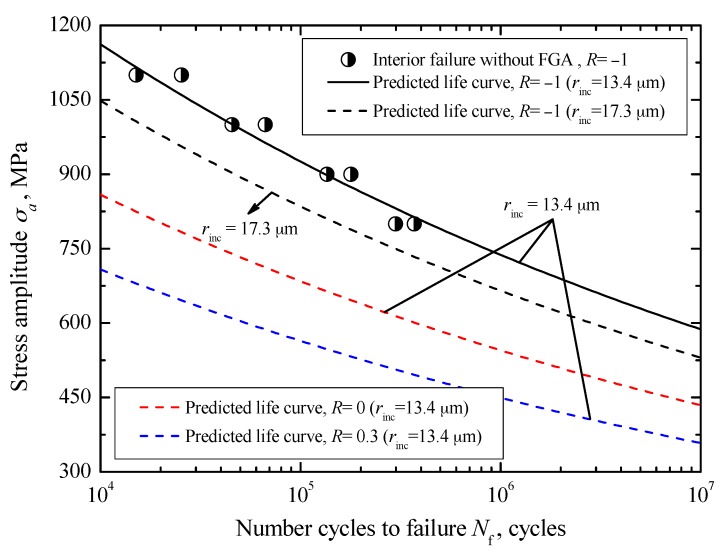
Predicted crack growth lives for interior failure without FGA.

For interior failure without FGA, combined with the sizes of inclusion and the relevant *S-N* data for *R* = −1, the values of *C*_int_ and *m*_int_ in Equation (24) were evaluated to be 3.75 × 10^−25^ and 15.73 respectively. Corresponding to the mean value and the maximum value of *r*_inc_, about 13.4 μm and 17.3 μm, the predicted crack growth life curves for *R* = −1 are indicated by a solid line and a dashed line in Figure 14, respectively. It can be found that the predicted curves all exhibit a continuously descending trend, which is consistent with the variation trend of the experimental data. Especially, the predicted curve associated with the maximum inclusion size indicates well the lower boundary of the data. Based on this, and by using Equation (25), the crack growth curves associated with the mean inclusion size under stress ratios of 0 and 0.3 is plotted by a red dashed line and a blue dashed line in Figure 14, respectively.

**Figure 15:**
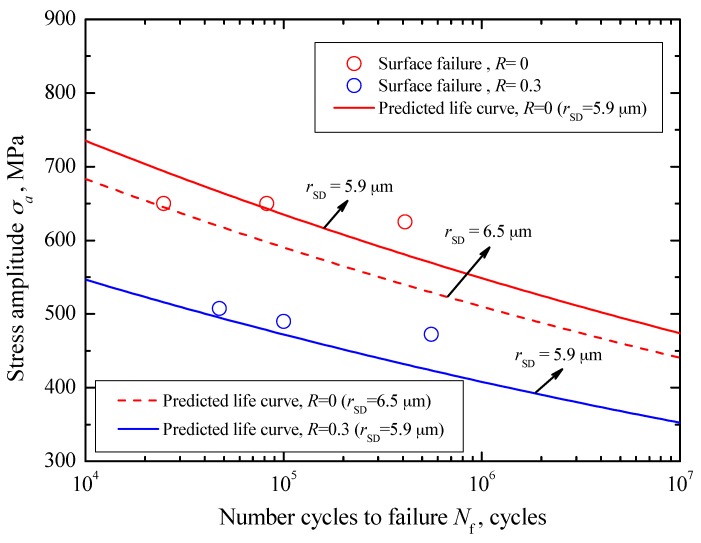
Predicted crack growth lives for surface failure.

For surface failure, combined with the sizes of SD and the relevant *S-N* data for *R* = 0, the values of *C*_sur_ and *m*_sur_ in Equation (26) were evaluated to be 3.75 × 10^−23^ and 12.28, respectively. Corresponding to the mean value and the maximum value of *r*_SD_, about 5.9 μm and 6.5 μm, the predicted crack growth life curves for *R* = 0 are indicated by a solid line and a dashed line in Figure 15, respectively. Similarly, based on the evaluated results for *R* = 0, the crack growth life curve corresponding to the mean value of *r*_SD_ for *R* = 0.3 is plotted by a dotted line in Figure 15. They all reflect well the variation trend of the experimental data.

**Figure 16:**
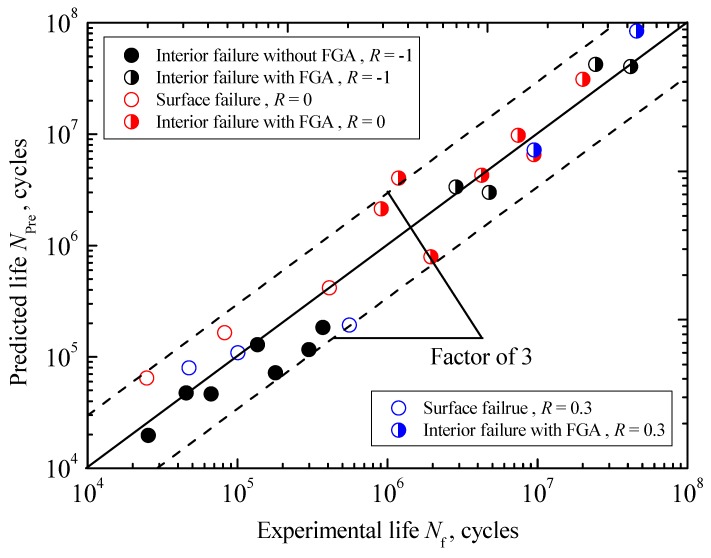
Comparison between the predicted and experimental results.

In addition, the comparison between predicted and experimental fatigue lives under three stress ratios is shown in Figure 16. It can be seen that the agreement is fairly good within a factor of three. Thus, it can be concluded that the proposed life prediction approaches, corresponding to the interior inclusion-FGA-fisheye induced failure governed by the interior crack initiation in the long-life regime, the interior inclusion-fisheye induced failure governed by the interior crack growth in the short life regime and the surface SD-SSA induced failure governed by the surface crack growth in the short life regime, are acceptable in engineering design.

## 4. Conclusions

The main conclusions obtained in this study are summarized as follows:

(1) Carburized Cr-Ni steel represents continuously descending *S-N* characteristics with interior inclusion-induced failure under *R* = −1, whereas it represents the duplex *S-N* characteristics with surface defect-induced failure and interior inclusion-induced failure under *R* = 0 and 0.3.
(2) The tensile stress tends to increase with increasing stress ratio, which overcomes the compressive residual stress and promotes crack initiation from surface defects or interior inclusions contained in the carburized layer.
(3) FGA formation not only depends on the number of loading cycles, but also is limited by the decreasing compressive stress and stress amplitude.
(4) The surface and interior failures in the short life regime below 5 × 10^5^ cycles can be characterized by crack growth process, while the interior failure with FGA in the long life regime beyond 10^6^ cycles can be characterized by the crack initiation process.
(5) The proposed life prediction models associated with interior inclusion-FGA-fisheye induced failure, interior inclusion-fisheye induced failure, and surface defect induced failure were validated by the good agreement between predicted and experimental results.
